# Reasons for the Early Introduction of Complementary Feeding to HIV-Exposed Infants in the Eastern Cape, South Africa: An Exploratory Qualitative Study

**DOI:** 10.3390/medicina56120703

**Published:** 2020-12-16

**Authors:** Daniel Ter Goon, Anthony Idowu Ajayi, Oladele Vincent Adeniyi

**Affiliations:** 1 Department of Public Health, University of Fort Hare, 5 Oxford Street, East London 5201, South Africa; oadeniyi@wsu.ac.za or; 2 Population Dynamics and Sexual and Reproductive Health, African Population and Health Research Centre, APHRC Campus, Manga Close, Nairobi 00100, Kenya; ajayianthony@gmail.com or; 3 Sociology Department, University of Fort Hare, East London 5201, South Africa; 4 Department of Family Medicine, East London Hospital Complex, Cecilia Makiwane Hospital, East London 5206, South Africa

**Keywords:** infant feeding, breastfeeding, mother-to-child HIV transmission, supplementary feeding

## Abstract

Exclusive breastfeeding has many health benefits for the baby and the mother. This study explored the reasons for the early introduction of supplementary feeding before six months, and the issues faced by parturient women in practicing exclusive breast feeding (EBF) for their HIV-exposed infants in the Eastern Cape, South Africa. Narratives from 319 parturient women with HIV (aged 18 years and above) were collected at three hospitals in the Eastern Cape through semi-structured interviews over a period of five months. Qualitative data were analysed using thematic content analysis. The maternal perception of HIV transmission from breast milk influenced the decision for the immediate introduction of formula feeding. Breast sores, lumps, surgery and perceived insufficiency of milk influenced the decision of mothers to initiate formula feeding within the first two months. However, mothers who initiated complementary feeding after two months were driven by factors common among newborns (refusal of breast milk, baby crying inconsolably and fear of losing weight) and social factors (economic or financial hardships and work-related challenges). Additionally, advice from family members weighed heavily in the decision to switch to complementary feeding, contrary to the healthcare providers’ recommendations. Early complementary feeding for HIV-exposed infants is influenced by maternal perceptions of breast milk transmission, breast and infant factors and socio-economic and cultural practices in the region. Thus, behavioural interventions tailored towards promoting exclusive breastfeeding practices in this population, starting from the pre-natal and continuing during the post-partum period, should also target the immediate family members. National policy should focus on creating an EBF-friendly environment at the workplace for women.

## 1. Introduction

The World Health Organization recommends the practice of exclusive breastfeeding (strictly breast milk with the exception of drugs) for infants in the first six months of life in low- and middle-income countries [1]. This is based on public health consensus that exclusive breastfeeding (EBF) in the first six months of life is the best for the optimal growth, development and survival of infants [2]. Breastfeeding should then continue for the next 24 months while carefully introducing safe, adequate and appropriate complementary foods from six months [1]. EBF has been proven to avert an estimated 13.8% of mortality in children below two years, globally [2]. Despite the adoption of the EBF policy and the universal availability of antiretroviral therapy for the prevention of mother-to-child transmission in South Africa [3], evidence suggests that the rate of EBF remains low. About 32% of mothers reported exclusively breastfeeding their infants between four and five months in South Africa [4]. Despite the concerted efforts of governmental and non-governmental agencies to promote EBF, certain barriers still persist and preclude its expansion. Identifying the barriers to the practice of EBF and the factors facilitating the early introduction of complementary feeding would be crucial for designing evidence-based policies and interventions that are context-specific.

Complementary feeding should be introduced at six months in order to optimize the nutritional status, growth, development and survival of a child, especially for HIV-exposed infants [2]. At six months, breast milk alone cannot meet all the nutritional requirements of the infants; therefore, complementary feeding becomes necessary [5]. Unfortunately, in both low- and middle-income countries including South Africa, there is an early introduction of complementary feeding by HIV positive mothers [6,7]. The rate of practice of complementary feeding changes over time; a recent study reported an early initiation rate of 13.1% (before six months), a timely initiation rate of 65.1% (at six months) and 21.8% late initiation (after six months) among mothers of HIV-exposed infants [8]. Early initiation of complementary feeding whilst still breastfeeding, otherwise referred to as mixed feeding, has been shown to be associated with serious adverse infant outcomes. Mebratu et al. [9] reported a relatively high rate (18.4%) of mixed feeding practices among mothers of HIV-exposed infants in Ethiopia.

Infants exposed to early complementary feeding (foods or liquids) whilst still breastfeeding are at risk of developing serious morbidity and, consequently, mortality from unsafe water, inadequate formula preparation or storage, unsanitary conditions and formula shortages [5]. In addition, other health problems including stunted growth, being underweight and wasting away are very common [6,10,11,12]. Infants who are exposed to early complementary feeding are more likely to experience dietary deficiencies due to the consumption of inappropriate nutritionally poor foods [13]. While the practice of early complementary feeding within the context of mixed feeding is deleterious for infants’ growth and development, the adverse outcomes are particularly worse for HIV-exposed infants. Studies have shown that HIV-exposed infants have a 1.5-fold increased risk for infectious morbidity compared to HIV unexposed infants, which is influenced by maternal antiretroviral treatment (ART) use and infant feeding, and restricted or no breastfeeding [14,15,16].

Evidence suggests that mothers living with HIV are more likely to introduce complementary feeding early (before six months) in comparison to HIV-negative mothers [7]. The reasons for these choices are varied; they include cultural and religious beliefs [7,17,18], the economic dependence of women and social support [7,19]. Other reasons include maternal age, HIV stigma, fear of HIV transmission and contradictory infant feeding counselling [7].

Since the Eastern Cape province has one of the highest burdens of HIV in South Africa [20], it is important to scrutinize facilitators of the early introduction of complementary feeding for HIV-exposed infants in order to tackle problems associated with malnutrition in these infants. Furthermore, exploring the feeding decisions of mothers is important for optimal improvement in child nutrition practice. Using an exploratory qualitative approach, this study explored the perspectives of mothers living with HIV on the reasons for the early introduction of complementary feeding to their HIV-exposed infants in the Eastern Cape, South Africa.

## 2. Materials and Methods

### 2.1. Study Design and Settings

This exploratory qualitative study forms part of the follow-up of the bigger study, “East London Prospective Cohort Study”. This cohort enrolled parturient women with HIV drawn from three large maternity centres (Frere, Cecilia Makiwane and Bisho Hospitals) in the Eastern Cape [21]. These hospitals provide maternal services for a combined population of approximately 1.7 million residents of the Buffalo City and Amathole districts. The data collection for this sub-study of parturient mothers was conducted from January to May 2018.

### 2.2. Participants

A total of 1709 mothers were recruited into the cohort between September 2015 and May 2016. However, only 485 parturient women of the East London Prospective Cohort Study were available for exit interviews. Participants were included in the follow-up study if they had been enrolled in the database and still had active telephonic contact. The majority of the participants could not be contacted telephonically and were excluded from the study. Parturient women who reported early introduction of complementary feeding (formula feeding or any other milk substitutes before six months) were included in this sub-study. Responses from 319 parturient women, who confirmed that they had initiated complementary feeding before six months to their infants, were analysed thematically in this sub-study. All mothers were on antiretroviral therapy, however, close to one in three women reported adherence challenges [22].

### 2.3. Data Collection Procedures and Tool

An interviewer-guided interview and a validated questionnaire were used to collect data from the participants. We recruited participants via telephone. Participants made the decision to either visit the nearest hospital for a contact interview or they consented to a telephonic interview. All interviews were conducted in English and IsiXhosa (local language) and were audio-recorded. Open-ended questions (semi-structured) were used to elicit the participants’ views on the underlying reasons for the early introduction of supplementary feeding to their infants. Participants were asked to describe how they fed their infants in the first six months after birth. Those who introduced other feeds beside EBF within six months were further probed to elicit the reasons for their decisions.

The following questions were asked:

- How did you feed your baby in the first six months after birth? (screening question)
- Since you introduced supplementary feeding early for your baby, what informed your decision(s) to feed your baby with the method you adopted?
- Can you tell me more about the challenges you experienced with practising exclusive breastfeeding for your baby?
- What benefits did you derive from feeding your baby with other products besides breast milk?
- What were your concerns about exclusive breastfeeding in the first six months after birth? (probing question, if details were not forthcoming).

### 2.4. Ethics Approval and Consent to Participate

The study received ethical approval from the Health Sciences’ Research Ethics Committee, Walter Sisulu University (ref: 085/2017). Permission was obtained from the Eastern Cape Provincial Department of Health Ethics Research Committee (EC_2015RP19_131) and the management of the selected hospitals prior to data collection. The aim and nature of the study were explained to all the participants and they gave informed consent to participate in the study.

### 2.5. Data Analysis

Qualitative data were analysed using the thematic content analysis method according to the steps described by Creswell [23]. The transcribed text of the interviews was translated verbatim and cross-checked for accuracy by a native IsiXhosa speaker. The notes were read repeatedly and the responses of the participants were organised into themes. All relevant information was clustered together and coded. After grouping the key issues, the notes were further double-checked to ensure the accuracy of the responses in their clusters. Secondly, emerging sub-themes on the reasons for the early introduction of complementary feeding were grouped. Finally, a reflexive review of the themes and sub-themes was conducted to construct a narrative on how parturient women with HIV had introduced solids and other breast milk substitutes to their exposed infants within the first 6 months. In order to ensure the credibility of the findings, transcripts of 10 interviews were randomly sampled and reviewed by the investigators.

## 3. Results

Participants were predominantly above 30 years old (70.2%), single (68.7%) and had attained grade levels of education between 8–12 (74.3%) (Table 1). The highest proportion of the participants initiated formula feeding immediately after the birth of their newborns (42.6%) (Table 1).

### 3.1. Reasons for Introducing Complementary Feeding since Child Birth

For women who introduced complementary feeding early, at childbirth, or soon after birth, the fear of transmitting HIV to their babies, breast-related issues and the pressure to resume work/school motivated their decision.

#### 3.1.1. Fear of Transmitting HIV to the Baby (Perception about Breast Milk Transmission of HIV)

Most mothers who introduced complementary feeding at childbirth did so because of their fear of transmitting the infection to their babies. To them, preventing mother-to-child transmission is as simple as not breastfeeding the baby.

A mother who is in this category explained:

“I started formula feeding because I don’t want my baby to get infected” (29-year-old; she introduced formula feeding immediately after leaving the hospital).

#### 3.1.2. Issues with Breast and Breast Milk Flow (Breast Factors)

Some women introduced complementary feeding due to issues with their breasts that make breastfeeding impossible.

Below are some of the verbatim quotations supporting the findings:

“I underwent surgery because milk was blocked in my breast” (42-year-old; she introduced formula feeding at one month).

“My breast had sores” (25-year-old; she introduced formula feeding after 10 weeks).

“My breast didn’t produce milk” (19-year-old; introduced formula feeding at childbirth).

“My tits were small and my baby could not suck them” (22-year-old; introduced formula after a week).

Yet, some confessed that due to economic hardships, they often resorted to breastfeeding because they could not sustain the formula feeding on a consistent basis.

“I have to look for alternative ways to provide feeds, because I am poor” (20-year-old; introduced formula after 2 weeks).

#### 3.1.3. Resumption of Work or School

A few women had to introduce formula feeding gradually after a month so that the child would adapt to the mother’s absence when she returned to work or school.

“I left the baby with my mum to return to school” (21-year-old; introduced formula feeding after one month).

### 3.2. Reasons for Introducing Complementary Feeding after Two Months or More

The main reasons for introducing supplementary feeding after two months or more were: the baby crying because of not getting enough food, having to return to work or school, the baby losing weight, the influence of elders, and the baby not wanting breast milk.

#### 3.2.1. Baby Crying Unconsolably

For most women who introduced complementary feeding earlier than six months, they had done so because the baby was crying and they assumed that their babies were not being fed adequately as the breast milk alone appeared to not sufficiently satisfy the baby’s hunger.

A 28-year-old mother said:

“I feel bad when the baby is crying, and I know if I can give my baby something to drink or eat, it may help stop the crying” (introduced formula at 10 weeks).

Another woman (32-year-old) succinctly summarised her interpretation about her baby crying unconsolably:

“My baby was not getting enough milk and kept crying. I decided to give my baby formula at two months”.

#### 3.2.2. Returning to Work or School

Employed mothers have difficulty practicing exclusive breastfeeding because of work-related challenges. They have to resume work as stipulated in their leave notification. They have no option other than to leave the baby at home with other relatives. In some cases, they are compelled to stop breastfeeding due to the long hours at work and work-related travel.

“I introduced formula feeding because I had to return to work” (35-year-old; introduced formula after 3 months).

“The work runs from 7.00 am to 7.00 pm and how can I practice exclusive breastfeeding?” (26-year-old; introduced formula feeding at 2 months).

“Even if I could take the baby to work, the work ethics don’t allow babies in the work place” (30-year-old; introduced formula at two months).

#### 3.2.3. Baby Did Not Want Breast Milk

Some mothers indicated that their babies refused breast milk, and given that they had no other alternatives, formula feeding was introduced.

“Baby didn’t want breastmilk, she was vomiting” (23-year-old; introduced formula feeding after 2 months).

#### 3.2.4. Infant Weight Loss

Some of the mothers perceived the weight of the baby was being affected with only exclusive breastfeeding. Yet, others expressed fear of their baby losing weight if they resorted to exclusively breastfeeding the baby.

“She was losing weight and decided to stop breastfeeding” (34-year-old; started formula feeding at 3 months).

“I won’t only breastfeed my baby. I will also provide formula feeding. My baby will lose weight and his health also will be affected, somehow. I am afraid to go with breast milk, alone” (28-year-old; introduced formula feeding at 2 months).

#### 3.2.5. Influence of Family Members

Some of the mothers indicated that they had stopped exclusive breastfeeding because of the influence of elderly people or family members and friends. They were encouraged to practise mixed feeding.

“One of the elder sisters told me to stop exclusively breastfeeding. She said I’m punishing the baby. The baby cannot be strong, feeding only on breast milk” (25-year-old; introduced formula at 3 months).

#### 3.2.6. Health Providers’ Advice

The participants indicated that their decisions had been influenced by the advice and counselling they had received from health care providers on the influence of exclusive breastfeeding on the health of mother and child in the context of HIV.

The following statements affirmed this:

“The nurse told me that because I’m HIV-positive. I have to breastfeed the baby for the first six months, without giving any other food, or I could choose the option of formula feeding. She indicated that if I chose the first option (exclusive breastfeeding), and I took my HIV medication regularly, the risk of my baby getting HIV from me would be low” (27-year-old; introduced formula at 3 months).

## 4. Discussion

This present study explored the reasons for the introduction of complementary feeding before six months (early) among HIV-positive mothers in the Eastern Cape, South Africa. The evidence clearly showed that mothers often practised mixed feeding in an attempt to introduce complementary feeding within the first six months of life [9,10,12]. To navigate the challenge of ensuring optimal feeding amidst concerns of HIV transmission from the breastmilk, the World Health Organization has advocated strongly that mothers and/or their infants should take a course of antiretroviral drugs to achieve viral suppression [24]. Mothers of HIV-exposed infants were advised to exclusively breastfeed for six months, then introduce solid foods, but continue breastfeeding for up to 24 months. Infant exclusive breastfeeding from birth to six months would ensure the optimal growth and development of the child [25]. Therefore, efforts to encourage and promote EBF among lactating mothers with HIV should include them refraining from introducing early supplementary foods; these efforts should be intensified to increase the EBF rates in the country.

Similarly, understanding the context-specific barriers of EBF is important in crafting interventions to tackle such barriers. This present study explored the reasons for the introduction of complementary feeding before six months among HIV-positive mothers in the Eastern Cape, South Africa. The findings indicate that, although the benefits of EBF had been clearly explained by the health care providers, mothers in this setting face challenges in achieving safer feeding practices. Most mothers introduced other foods to newborns at childbirth because of the mothers’ fears of transmitting infections to their babies. They felt that complementary feeding would eliminate the chances of transmission of HIV to their babies. As echoed by a mother: “I started formula feeding because I don’t want my baby to get infected”. This finding resonates with studies conducted in Zambia, South Africa and Malawi [26,27,28], which indicate that mothers feared their babies may be infected with HIV through breast milk. This implies that mothers are shackled by the breast milk transmission risks to their infants. The protective effect of antiretroviral therapy should form the core of education of mothers at postnatal wards and clinics in order to promote safe breastfeeding in the context of maternal HIV in the region and the country.

Maternal and infant factors affecting early breastfeeding cessation were breast sores or lumps, surgery, perceived insufficiency of milk, the baby refusing to suck the breast, the baby crying, and the baby losing weight. The findings of the present study concur with several studies conducted in South Africa, UK, Tanzania, Kenya, Singapore and Bangladesh [27,28,29,30,31,32,33,34]. The practice of providing liquids or solids other than breast milk before six months contradicts WHO recommendations for infant feeding [11]. Therefore, clinicians should enquire and address concerns relating to breast factors during the post-natal visits in order to mitigate the chances of mothers stopping EBF.

Another finding was related to economic or financial hardships influencing women to explore alternative feeding options. Given the high rate of unemployment (64%) and single status (68.7%) reported in this study, it is surprising that some of the women reported economic hardships as the reasons for the early initiation of complementary feeding in this population. This further supports the AFASS—Acceptable, Feasible, Affordable, Sustainable, and Safe—criteria for guiding mothers during the prenatal period on their future decision to elect formula feeding in low resource settings [24]. Women who cannot sustain formula feeding often mix-feed, which is a recipe for severe malnutrition and gastroenteritis [24]. As such, the feeding practices of mothers should be monitored consistently during the post-natal period, especially during the first six months.

Yet another reason for stopping breast milk at two months was work/school-related challenges. The mothers indicated that they had to resume work, long hours of work, work travel engagements, and were faced with a lack of support in the workplace for breastfeeding. Several studies have reported work-related challenges as one of the underlying factors affecting EBF for the first six months among HIV-positive mothers in South Africa [27,35]. Other studies conducted elsewhere have also indicated work-related issues affecting the EBF of working mothers [33,36]. Some of the mothers in this present study chose the option of providing infants with supplementary foods for them to resume work. In fact, some women started to introduce formula feeding gradually before two months in preparation for an early return to work. In South Africa, workplace facilities are not supportive of exclusive breastfeeding practice. The four months’ maternity leave for women working in government organisations appears insufficient to aid the practice of EBF. Thus, this policy should be revised to reflect six months of EBF, and workplace environments should provide the necessary support and promote exclusive breastfeeding.

Some of the mothers indicated that they had stopped exclusive breastfeeding because of the influence of elderly people or family members and friends. Mothers are likely to either adopt or reject the traditional practices related to infant nutrition which are provided by elders, usually the grandmothers. This tends to suggest that family members and friends have some control on an infant’s nutrition. The experiences and beliefs of some family members that human milk alone is not adequate for the infant still occur; therefore, an introduction to solid foods from birth cannot be ruled out [37,38]. Studies from low-income countries have reported culturally-inclined advice from grandmothers as influencing the pre-lacteal feeding of infants [39,40,41,42]. Given that cultural practices have an influence on EBF practice, it is imperative that family members be included in breastfeeding educational programmes. This strategy has proven to be successful in improving EBF practice in other countries, including Brazil, Thailand and China [43,44,45].

The participants indicated that they received advice or counselling from health care providers on exclusive breastfeeding concerning the health of the mother and child in the context of HIV. However, the messages received from family sources were contradictory and they conflicted with the health professionals’ advice. The health professionals advised and emphasised EBF for the first six months, without giving any solids or other liquids to the infant. On the other hand, the family would simply advise the mothers to jettison the concept of EBF, indicating that breast milk ‘only’ is not enough for the infant’s healthy growth. Of course, this incongruence or conflicting stance on EBF between health providers and family members is expected. Apparently, health providers’ counselling is centred on a biomedical and health perspective, while the family advice is traditionally-inclined and based on own past experiences. Again, this is a pointer to the fact that educational intervention should not only focus on the mothers at antenatal clinics, but should also target family members on the importance and benefits of providing EBF [46].

## 5. Implications

Our findings highlight the reasons for the early introduction of supplementary feeding before six months, and the challenges faced by parturient women in practicing EBF on their HIV-exposed infants in the Eastern Cape, South Africa. Breast sores, lumps, surgery and perceived insufficiency of milk are influencers of the decision to initiate formula feeding within the first two months. The factors responsible for initiating supplementary feeding after two months were the infants’ refusal of breast milk, the baby crying uncontrollably, fear of the baby losing weight, economic or financial hardships, work-related challenges and the advice of family members. These findings suggest that behavioural interventions should not only focus on mothers but should also target their immediate family members. In addition, there is a need to create an EBF-friendly environment at the workplace for women.

## 6. Limitations

Our study was limited to parturient mothers with HIV-exposed infants in selected hospitals in the Eastern Cape Province; therefore, more studies are needed to explore other settings. Notwithstanding, the findings of this study provide considerable insight into the mothers’ experiences regarding EBF, at least in this setting, and they create an opportunity for critical reflection.

## 7. Conclusions

The findings from this study indicate that the fear of HIV transmission to the baby, maternal-infant factors (breast sores or lumps, surgery, perceived insufficiency of milk, the baby refusing the breast, the baby crying and the baby losing weight), economic or financial hardships and work-related challenges negatively influence the infant feeding practices of parturient women with HIV in this setting. Additionally, mothers received advice or support from family members and healthcare providers, which shapes their choice to continue or stop EBF practice. Behavioural interventions should not only focus on mothers but also target their immediate family members, and create an EBF-friendly environment at the workplace for women.

## Figures and Tables

**Table 1:** Demographic information of respondents.

Variables	Did Not Breastfeed Exclusively for Six Months *n* = 319	Percentage
Age		
24 years and less	22	6.9
25–29 years	73	22.9
30–34 years	96	30.1
35–39 years	85	26.6
40 years and above	43	13.5
Marital status		
Single	219	68.7
Married	79	24.8
Cohabiting	16	5.02
Previously married	5	1.6
Education level		
Grade 7 and less	18	5.6
Grade 8–12	237	74.3
Higher education	25	7.8
Employment status		
Unemployed	204	64.0
Employed	115	36.0
Timing of initiation of supplementary feeding		
After birth	136	42.6
One	50	15.7
Two	41	12.9
Three	44	13.8
Four	38	11.9
Five	10	3.1
